# ﻿*Balitoraanlongensis*, the first cavefish species of the genus *Balitora* (Teleostei, Balitoridae) from Guizhou Province, southwest China

**DOI:** 10.3897/zookeys.1185.108545

**Published:** 2023-11-28

**Authors:** Tao Luo, Zhi-Xia Chen, Xin-Rui Zhao, Jing Yu, Chang-Ting Lan, Jia-Jun Zhou, Ning Xiao, Jiang Zhou

**Affiliations:** 1 School of Karst Science, Guizhou Normal University, Guiyang 550001, Guizhou, China; 2 School of Life Sciences, Guizhou Normal University, Guiyang 550025, Guizhou, China; 3 Zhejiang Forest Resource Monitoring Center, Hangzhou 310020, Zhejiang, China; 4 Zhejiang Forestry Survey Planning and Design Company Limited, Hangzhou 310020, Zhejiang, China; 5 Guiyang Healthcare Vocational University, Guiyang 550081, Guizhou, China

**Keywords:** Nanpanjiang River, stone loach, taxonomy, phylogeny

## Abstract

This work describes a new species, *Balitoraanlongensis***sp. nov.**, collected from a cave at Xinglong Town, Anlong County, Guzihou, China. Phylogenetic trees reconstructed based on two mitochondrial and three nuclear genes show that the new species represents an independent evolutionary lineage with large genetic differences, 7.1%–12.0% in mitochondrial gene cytochrome *b* and 9.2%–12.1% in cytochrome oxidase subunit 1, from congeners. Morphologically, the new species can be distinguished from the 18 species currently assigned to the genus *Balitora* by a combination of characters, most clearly by having two pairs of maxillary barbels; 8½ branched dorsal-fin rays; 5½ branched anal-fin rays; pectoral fin not reaching pelvic fin origin; dorsal-fin origin in front of pelvic fin origin; eye small (eye diameter approximately equal to outer maxillary barbel length); and fins lacking pigment in live fish. The new species represents the first record of *Balitora* inhabiting caves in China and increases the number of species in the genus *Balitora* in its present concept from 18 to 19. The study suggests that more evidence is needed to further clarify the taxonomic composition of the genus *Balitora*.

## ﻿Introduction

The karst region of southwest China is well known as a distinctive center and hotspot of biodiversity (Myers et al. 2000). Distinctive karst habitats have created varied natural landscapes and organisms, such as cave river ecosystems and the organisms that inhabit them. Cavefishes are typical cave organisms (Niemiller et al. 2019). In China, there are approximately 170 endemic species of cavefishes belonging to two orders (Cypriniformes and Siluriformes) and four families (Cyprinidae, Nemacheilidae, Cobitidae, and Amblycipitidae) (Ma et al. 2019). In previous reports, cavefishes were mainly recorded in the Cyprinidae (83 species), Nemacheilidae (77 species), and Cobitidae (eight species) (Ma et al. 2019) (Suppl. material 1). New discoveries may be possible at the family, genus, and species level, considering that past surveys and reports of cavefishes have mainly focused on Guangxi and Yunnan provinces, China (Ma et al. 2019).

The genus *Balitora*, Gray, 1830 was established with *Balitorabrucei* as the type species, originally placed in the Cobitidae (Gray, 1830), and is now placed within the Balitoridae (Fricke et al. 2023). *Hemimyzon* Regan, 1911 and *Sinohomaloptera* Fang, 1930, which are taxonomically closely related to *Balitora*, were established using *H.formosanum* and *S.kwangsiensis*, respectively, as the type species. The genus *Balitora* has long been the subject of taxonomic controversy, with different taxonomic schemes proposed based on morphological differences. Chen (1978) recognized one pair of maxillary barbels as a character that distinguishes *Balitora* from other genera. Later, several new *Balitora* species were reported, including *B.pengi* Huang, 1982, *B.tchangi* Zheng, 1982, *B.nujiangensis* Zhang & Zheng, 1983, and *B.elongata* Chen & Li, 1985 (Zheng et al. 1982; Zheng and Zhang 1983; Li and Chen 1985), which are currently placed in the genus *Hemimyzon* (Kottelat and Chu 1988). Kottelat and Chu (1988) and Kottelat (1988) reviewed the genus and considered *Sinohomaloptera* to be a synonym of *Balitora* as having one or two pairs of maxillary barbels. Based on three or more unbranched pelvic fin rays, *B.pengi*, *B.tchangi*, *B.nujiangensis*, and *B.elongata* were placed in *Hemimyzon*, while species with two branched pelvic fin rays were placed in *Balitora*, namely *B.lancangjiangensis* (Zheng, 1980), *B.kwangsiensis* (Fang, 1930), and *B.longibarbata* (Chen, 1982) (Kottelat and Chu 1988). Chen (1990) accepted this suggestion for a taxonomic revision of the species distributed in Yunnan. However, the suggestion of Kottelat and Chu (1988) was not adopted by Chinese scholars, and species were still placed in *Balitora* based on the number of maxillary barbel, and *Sinohomaloptera* was considered valid (Chen and Tang 2000; Jiang et al. 2016). Nevertheless, previous morphology-based studies have not resolved the phylogenetic relationships between *Balitora*, *Hemimyzon* and *Sinohomaloptera* due to a lack of molecular evidence.

Few molecular markers have been used to assess the phylogeny of the genus *Balitora*. A phylogenetic tree reconstructed by Šlechtová et al. (2007) based on the nuclear gene RAG1 showed that a species identified as Balitorasp.cf.burmanica showed that the genus *Balitora* is nested within the genus *Homaloptera*. The phylogeny of Liu et al. (2012a) based on mitochondrial (COI and ND4+ND5) and nuclear genes (RH1, RAG1, EGR2B, and IRBP), on the other hand, supports the view that *Balitora* (*S.kwangsiensis*) is close to *Sinogastromyzon*. Kumkar et al. (2016a) used two mitochondria (COI and Cyt*b*) to show for the first time phylogenetically that *Balitora* is not a monophyletic, but can be divided into three major clades. Keskar et al. (2018) then supported *Balitora* as a sister clade of *Lepidocephalichthys* based on mitochondrial COI and Cyt*b.*Tao et al. (2019) recovered *Balitora* (only *B.elongata*) as a sister clade of *Sinohomaloptera* (only *S.kwangsiensis*) based on a large-scale phylogeny of one mitochondrial and 14 nuclear genes. More recently, a phylogeny based on the mitochondrial genome (only *B.ludongensis* Liu & Chen, 2012) strongly support *Balitora* being close to ((*Jinshaia* + *Lepturichthys*) + *Sinogastromyzon*) (Shao et al. 2020). Based on the above various studies, it can be concluded that the phylogenetic position of the genus *Balitora* is unclear and may not be monophyletic.

To date, the classification of the genus remains controversial, mainly because of the lack of clear phylogenetic relationships and stable morphological characters among *Balitora*, *Hemimyzon*, and *Sinohomaloptera* (Table 1). In this study, we followed the latest taxonomic scheme after a comprehensive review, and *Balitora* was recorded with 19 species (Table 1), of which ten species are distributed in China, namely *B.brucei* Gray, 1830, *B.burmanica* Hora, 1932, *B.elongata*, *B.kwangsiensis*, *B.lancangjiangensis*, *B.longibarbata*, *B.ludongensis*, *B.nantingensis* Chen, Cui & Yang, 2005, *B.meridionalis* Kottelat, 1988, and *B.tchangi* (Liu et al. 2012b; Jiang et al. 2016) (Table 1). However, because this work did not have access to the original data for the four species (*B.haithanhi*, *B.nigrocorpa*, *B.vanlani*, and *B.vanlongi*) described by Nguyen (2005), as well as because *B.haithanhi* Nguyen, 2005, *B.nigrocorpa* Nguyen, 2005, and *B.vanlani* Nguyen, 2005 were used as synonyms of *B.kwangsiensis* and *B.vanlongi* was used as a synonym of *B.lancangjiangensis* (Kottelat 2012, 2013), considering that there are no significant morphological differences between them, together with the fact that these four species are from Vietnam, they are unlikely to be conspecific (Bhoite et al. 2012). Thus, these four species were excluded from the diagnosis of the new species (Conway and Mayden 2010; Bhoite et al. 2012; Liu et al. 2012a). To date, no cave species have been discovered within the genus *Balitora*.

**Table 1.** Taxonomic revision history of 19 species of the genus *Balitora* distributed in Asia. Species in bold are those which have a distribution in China.

ID #	Species	Kottelat and Chu 1988a	Chen and Tang 2000	Kottelat 2012	Jiang et al. 2016	Zhang and Zhao 2016	Fricke et al. 2023	Zhang and Cao 2021	This study
1	*Balitoraannamitica* Kottelat, 1988	–	–	*Balitora*	–	–	*Balitora*	–	*Balitora*
2	***Balitorabrucei* Gray, 1830**	–	–	*Balitora*	Balitora	–	*Balitora*	*Balitora*	*Balitora*
3	***Balitoraburmanica* Hora, 1932**	–	–	*Balitora*	Balitora	–	*Balitora*	*Balitora*	*Balitora*
4	*Balitorachipkali* Kumkar, Katwate, Raghavan & Dahanukar, 2016	–	–	–	–	–	*Balitora*	–	*Balitora*
5	*Balitoraeddsi* Conway & Mayden, 2010	–	–	*Balitora*	–	–	*Balitora*	–	*Balitora*
6	***Balitoraelongata* Chen & Li, 1985**	*Hemimyzon*	*Balitora*	*Hemimyzon*	*Balitora*	*Balitora*	*Hemimyzon*	*Balitora*	*Balitora*
7	*Balitorahaithanhi* Nguyen, 2005	–	–	? *Balitorahaithanhi*	–	–	*Balitora*	–	*Balitora*
8	*Balitorajalpalli* Raghavan, Tharian, Ali, Jadhav & Dahanukar, 2013	–	–	–	–	–	*Balitora*	–	*Balitora*
9	***Balitorakwangsiensis* (Fang, 1930)**	*Balitora*	–	*Balitora*	*Balitora*	*Balitora*	*Balitora*	*Balitora*	*Balitora*
10	***Balitoralancangjiangensis* (Zheng, 1980)**	*Balitora*	*Balitora*	*Balitora*	*Balitora*	*Balitora*	*Balitora*	*Balitora*	*Balitora*
11	*Balitoralaticauda* Bhoite, Jadhav & Dahanukar, 2012	–	–	*Balitora*	–	–	*Balitora*	–	*Balitora*
12	***Balitoralongibarbata* (Chen, 1982)**	*Balitora*	–	*Balitora*	*Balitora*	*Balitora*	*Balitora*	*Balitora*	*Balitora*
13	***Balitoraludongensis* Liu & Chen, 2012**	–	–	*Balitora*	*Balitora*	*Balitora*	*Balitora*	*Balitora*	*Balitora*
14	***Balitorameridionalis* Kottelat, 1988**	–	–	*Balitora*	*Balitora*	–	*Balitora*	*Balitora*	*Balitora*
15	*Balitoramysorensis* Hora, 1941	–	–	*Balitora*	–	–	*Balitora*	–	*Balitora*
16	***Balitoranantingensis* Chen, Cui & Yang, 2005**	–	–	*Balitora*	*Balitora*	–	*Balitora*	*Balitora*	*Balitora*
17	*Balitoravanlani* Nguyen, 2005	–	–	? *Balitoravanlani*	–	–	*Balitora*	–	*Balitora*
18	*Balitoravanlongi* Nguyen, 2005	–	–	? *Balitoravanlongi*	–	–	*Balitora*	–	*Balitora*
19	***Balitoratchangi* Zheng, 1982**	*Hemimyzon*	*Balitora*	*Hemimyzon*	*Balitora*	*Balitora*	*Hemimyzon*	*Balitora*	*Balitora*

In January 2023 during a survey of cavefishes in southwestern Guizhou Province, upper Pearl River, China, a number of specimens of the genus *Balitora* were collected, identified by two pairs of maxillary barbels. Morphological examination and molecular phylogenetic analysis suggested that these specimens can be distinguished from all other species presently assigned to the genus *Balitora*. Here, we describe these specimens as a new species, *Balitoraanlongensis* sp. nov.

## ﻿Materials and methods

### ﻿Sampling

**Figure 1.**
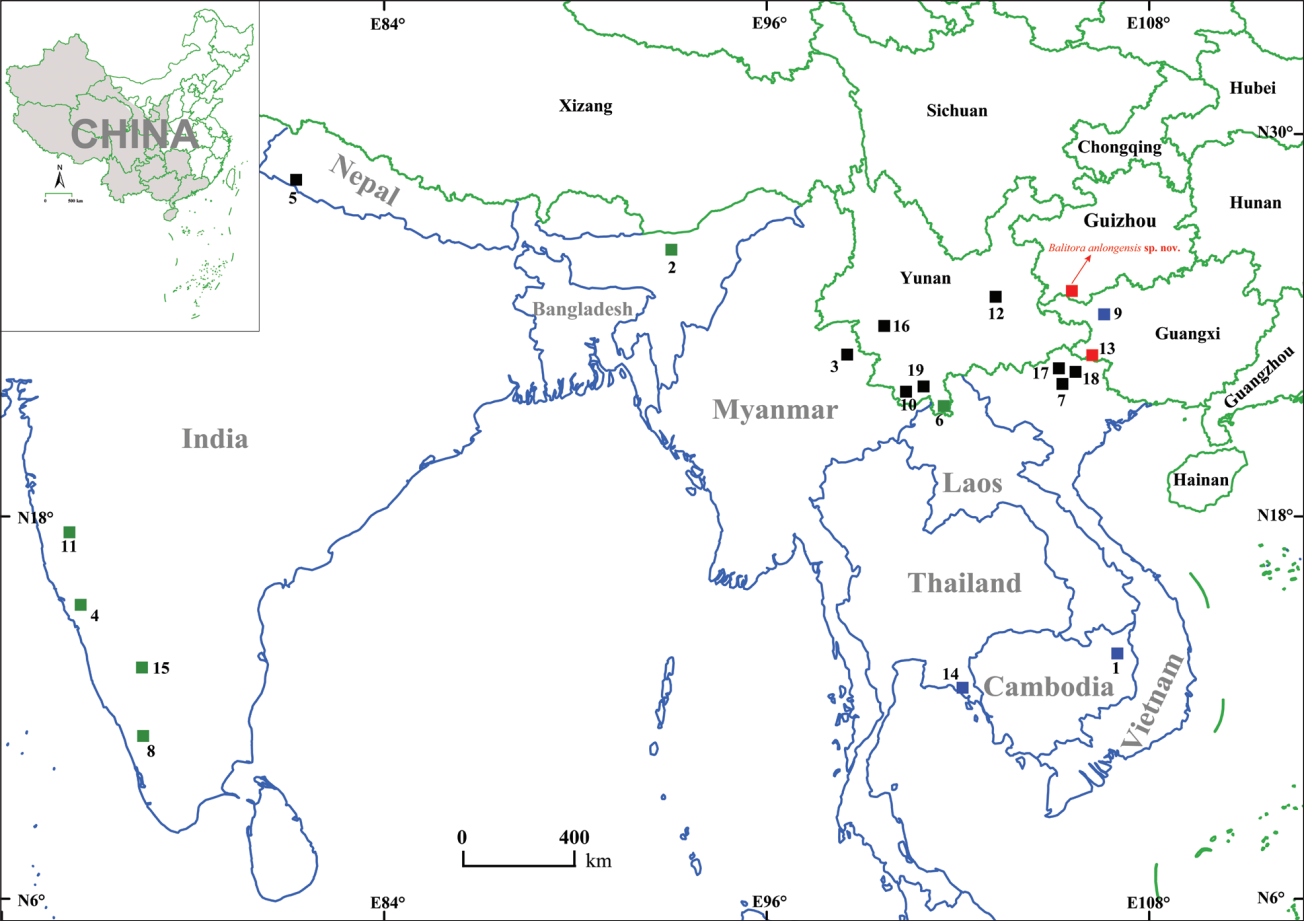
Sampling collection location of *Balitoraanlongensis* sp. nov. and distribution of 21 species of the genus *Balitora* (including *B.haithanhi*, *B.nigrocorpa*, *B.vanlani*, and *B.vanlongi*) in Asia. Species codes are provided in Table 1.

**Figure 2.**
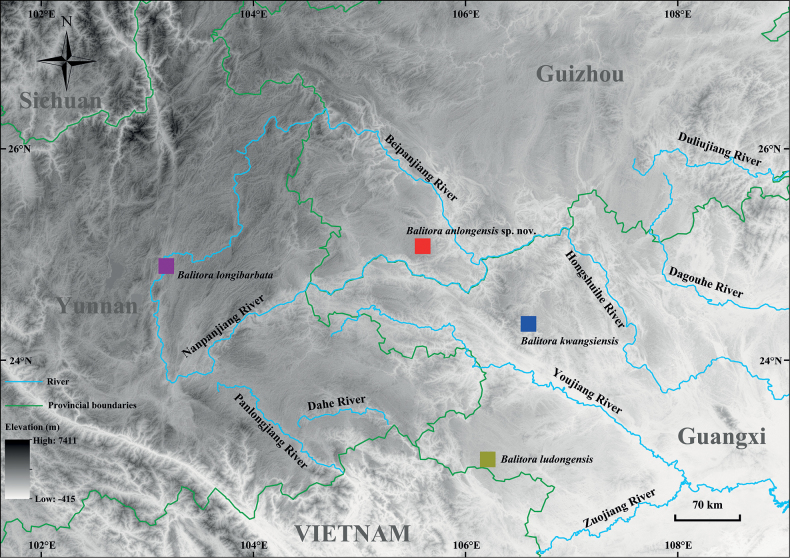
Distribution and hydrology of the type locality of the new species *Balitoraanlongensis* sp. nov. (red), *B.kwangsiensis* (blue), *B.longibarbata* (purple), and *B.ludongensis* (red), in the Pearl River basin, Southwest China.

This work collected a total of 30 specimens from three species for morphological comparison and genetic analysis (Figs 1, 2). Among these specimens, 11 specimens representing a new species, *Balitoraanlongensis* sp. nov., were from Xinglong Town, Anlong County, Guzihou, China; two specimens were *B.ludongensis* from Ludong Town, Jingxi County, Guangxi, China; and three specimens were *B.kwangsiensis* from Amojiang River basin, Yunnan, China. All specimens were fixed in 10% buffered formalin and later transferred to 75% ethanol for preservation. Muscle samples used for molecular analysis were preserved in 95% alcohol and stored at −20 °C. All specimens were kept at Guizhou Normal University, Guiyang City, Guizhou Province, China.

### ﻿DNA extraction, polymerase chain reaction (PCR), and sequencing

Genomic DNA was extracted from muscle tissue using a DNA extraction kit from Tiangen Biotech Co., Ltd. (Beijing, China). Five tissue samples used for molecular analysis were amplified and sequenced for two mitochondrial gene and three nuclear genes: mitochondrial gene cytochrome *b* (Cyt*b*), cytochrome oxidase subunit 1 (COI), recombination activating gene 1 (RAG1), interphotoreceptor retinoid binding protein (IRBP), and early growth response protein 2B (EGR2B). Primer sequences are shown in Suppl. material 2. PCR amplifications were performed in a 20-μl reaction volume with the following cycling conditions: an initial denaturing step at 95 °C for 4 min, 35 cycles of denaturing at 95 °C for 30 s, annealing at 52 °C for 1 min, and extension at 72 °C for 1 min, followed by a final extension step at 72 °C for 10 min. The PCR products were puriﬁed with spin columns. The products were sequenced on an ABI Prism 3730 automated DNA sequencer at Chengdu TSING KE Biological Technology Co., Ltd. (Chengdu, China). All newly obtained sequences have been submitted to GenBank (Table 2).

**Table 2.** Localities, voucher information, and GenBank numbers for all samples used in this study. Bold sequences are new from this study.

ID	Family/ species	Locality (* type localities)	Voucher	Cyt *b*	COI	RAG1	IRBP	EGR2B
	Balitoridae							
1	*Hemimyzonyaotanensis*	Mudong, Chongqing City, China	IHB0809019	JN176994	–	JN177053	–	–
2	*Sinogastromyzonszechuanensis*	Neijiang, Sichuan Province, China	20170920BB03	MN241814	MN241814	–	–	–
3	*Sinogastromyzonhsiashiensis*	Taoyuan, Hunan Province, China	IHB1004171/IHB030105	JN176997	JN177109	JN177054	–	–
4	*Lepturichthysdolichopterus*	Jianou, Fujian Province, China	IHB0706070	GU084245	JN177091	JN177029	–	–
5	*Lepturichthysfimbriata*	Jinkou, Hubei Province, China	IHB0803128	GU084229	JN177102	JN177182	JN177272	JN177251
6	*Sinogastromyzonnantaiensis*	Taiwan Province, China	ASIZP0806662	–	KU943003	–	–	–
7	*Sinogastromyzonpuliensis*	–	–	FJ605359	FJ605359	–	–	–
8	*Metahomalopteraomeiensis*	Mudong, Chongqing City, China	IHB0301071	JN177000	JN177080	JN177041		
9	*Sinogastromyzonsichangensis*	Cishui, Guizhou Province, China	IHB0400184	JN176998	JN177078	JN177040	KP695068	KP694496
10	*Sinogastromyzonsichangensis*	–	P4	OQ754146	–	OQ754160	OQ754156	OQ754151
11	*Jinshaiasinensis*	Chongqing City, China	IHB0301068	JN176985	JN177117	JN177044	–	–
12	*Jinshaiaabbreviata*	Guizhou Province, China	IHB0709424	JN176992	JN177228	JN177180	JN177274	JN177249
13	*Lepturichthysfimbriata*	Mudong, Chongqing City, China	IHB0301070	JN176942	JN177139	JN177020	JN177273	–
14	*Hemimyzonformosanus*	–	cyp903	AY392484	KU943001	–	KP695096	–
15	*Hemimyzontaitungensis*	Taiwan Province, China	–	KX056121	–	–	–	–
16	*Balitoraludongensis*	Jingxi City, Guangxi Province, China	GZNU20230215023	**OQ754141**	–	–	–	–
17	*Balitoraludongensis*	Jingxi City, Guangxi Province, China	GZNU20230215024	**OQ754142**	–	–	–	–
18	*Balitoraludongensis*	Jingxi City, Guangxi Province, China	SCAU-20190805001	MT157616	MT157616	–	–	–
19	*Balitoraanlongensis* sp. nov.	Xinglong Town, Anlong County, Guzihou, China*	GZNU20230215018	**OQ754144**	**OQ784688**	**OQ754158**	**OQ754154**	**OQ754149**
20	*Balitoraanlongensis* sp. nov.	Xinglong Town, Anlong County, Guzihou, China*	GZNU20230215019	**OQ754145**	**OQ784690**	**OQ754159**	**OQ754155**	**OQ754150**
21	*Balitoraanlongensis* sp. nov.	Xinglong Town, Anlong County, Guzihou, China*	GZNU20230215020	**OQ754143**	**OQ784689**	**OQ754157**	**OQ754152**	**OQ754148**
22	*Sinogastromyzonwui*	Zhaoping, Guangxi Province, China	IHB0400321	JN177001	JN177076	–	–	–
23	*Hemimyzonnujiangensis*	Nujiang, Yunnan Province, China	/	–	KM610757	–	–	–
24	*Hemimyzonnujiangensis*	Nujiang, Yunnan Province, China	ihb201305588	–	KM610756	–	–	–
25	*Sinogastromyzontonkinensis*	Yuanjiang, Yunnan Province, China	IHB0805543	JN177002	JN177074	JN177056	JN177277	JN177247
26	*Balitorabrucei*	–	CIFEFGB-Bb-02	MK732323	MK388804	–	–	–
27	*Balitorabrucei*	–	HSLBB	–	KJ774109	–	–	–
28	*Balitoraannamitica*	–	–	–	–	EF056359	–	–
29	*Balitoramysorensis*	India, Karnataka, Hattihole	WILD-15-PIS-231	KU378018	KU378005	–	–	–
30	*Balitoramysorensis*	India, Karnataka, Hattihole	BNHS FWF 197	KU378019	KU378006	–	–	–
31	*Balitorachipkali*	India, Karnataka, Ramnagar	BNHS FWF 193	KU378016	KU378003	–	–	–
32	*Balitorachipkali*	India, Karnataka, Kamra, Joida	WILD-15-PIS-230	KU378017	KU378004	–	–	–
33	*Balitorajalpalli*	/	KUFOS-19-AN-BA-34.1	–	MT216524	–	–	–
34	*Balitoralaticauda*	India, Maharashtra, Venegaon	WILD-12-PIS-019	KU378007	KU377994	–	–	–
35	*Balitoralaticauda*	India, Maharashtra, Venegaon	ZSI-WRC P/2849	KU378008	KU377995	–	–	–
36	*Balitoraelongata*	Menglun, Yunnan Province, China	IHB0301053	DQ105218	–	–	–	–
37	*Balitoraelongata*	Xishuangbanna, Yunnan Province, China	IHB0301030	DQ105217	–	–	–	–
38	*Balitorameridionalis*	–	–	–	–	KP322550		
39	*Balitorakwangsiensis*	Yuanjiang, Yunnan Province, China	IHB0805545	JN177004	–	JN177060	–	–
40	*Balitorakwangsiensis*		GZNU20230215022	**OQ754147**		**OQ754161**	**OQ754153**	–
41	*Balitoraelongata*	/	cyp74	–	–	KP695617	KP695065	–
42	*Balitorakwangsiensis*	Yuanjiang, Yunnan Province, China	IHB0805546	JN177006	JN177071	JN177058		
43	*Balitorakwangsiensis*	Yuanjiang, Yunnan Province, China	IHB0805547	JN177005	JN177072	JN177059	JN177276	JN177248
44	*Balitorakwangsiensis*	/	/	DQ105216	–	–	–	–
45	*Homalopterabilineata*	–	–	–	–	KP322549	–	–
46	*Homalopteraconfuzona*	–	CBM:ZF 11705	NC_033955	NC_033955	KP322543	–	–
47	*Homalopteraparclitella*	–	–	NC_031634	NC_031634	EU409610	EU409668	EU409732
48	*Homalopteraocellata*		CBM:ZF 12287	NC_033953	NC_033953	KP322539	–	–
49	*Homalopteraogilviei*	–	–	NC_031635	NC_031635	–	–	–
50	*Pseudohomalopteraleonardi*	–	–	AB242165	AB242165	EU711130	FJ197076	AB531164
51	*Pseudohomalopterasexmaculata*	–	–	–	ON903153	KP322545	–	–
52	*Balitoropsisophiolepis*	–	Vial 2006-0588	–	KR052868	KP322540	–	–
53	*Balitoropsiszollingeri*	–	Vial 2005-0948	–	KR052865	KP322535	–	–
54	*Bhavaniaaustralis*	–	KUFOS.2019.12.54	MT002547	MT002512	MT002571	–	–
55	*Homalopteramontana*	–	NBFGR:8118E	–	HQ219123	–	–	–
56	*Travancoriaelongata*	–	–	–	MT216535	–	–	–
57	*Travancoriajonesi*	–	KUFOS-19-AN-TR-49.1	–	MT216539	–	–	–
58	*Ghatsamontana*	–	KUFOS-19-RR-GA-39.1	–	MT216529	–	–	–
59	*Ghatsasanthamparaiensis*	–	KUFOS-19-AN-GA-42.1	–	MT216532	–	–	–
60	*Ghatsapillaii*	–	KUFOS-19-AN-GA-40.1	–	MT216530	–	–	–
61	*Homalopteroidessmithi*	–	CBM:ZF 12281	NC_033957	NC_033957	KP322546	–	–
62	*Homalopterulagymnogaster*	–	Vial SN25	–	–	KP322554	–	–
63	*Neohomalopterajohorensis*	–	CBM:ZF 12286	NC_033952	NC_033952	–	–	–
	Gastromyzontidae							
64	*Beaufortialiui*	Panzhihua, Sichuan Province, China	IHB1004172	JN177009	JN177069	–	–	–
65	*Beaufortiaszechuanensis*	Zhaotong, Yunna Province, China	IHB0709025	JN177007	JN177067	JN177061	JN177281	JN177253
66	*Formosaniachenyiyui*	Changting, Fujia Province, China	IHB0301051	MK135435	MK135435	–	–	–
67	*Vanmaneniacaldwelli*	Wuyishan, Fujian Province, China	IHB0706028	JN177011	JN177232	JN177178	JN177280	JN177252
	Catostomidae							
68	*Myxocyprinusasiaticus*	Mudong, Chongqing City, China	IHB0809033	JN176936	–	JN177063	–	JN177263

### ﻿Phylogenetic analyses

A total of 168 sequences (48 Cyt*b*, 55 COI, 36 RAG1, 18 IRBP, and 17 EGR2B sequences) was used for molecular analysis, including 30 newly sequenced sequences and 143 sequences downloaded from GenBank. This work followed the phylogenetic study reported by Kumkar et al. (2016a) and selected *Beaufortialiui* Chang 1944, *Beaufortiaszechuanensis* (Fang, 1930), *Formosaniachenyiyui* (Zheng, 1991), *Vanmaneniacaldwelli* (Zheng, 1991), and *Myxocyprinusasiaticus* (Bleeker, 1864) as outgroups.

All sequences were assembled and aligned using the MUSCLE (Edgar 2004) module in MEGA v. 7.0 (Kumar et al. 2016b) with default settings. The alignment results were checked by eye. The sequences after alignment and check were combined using PhyloSuite v. 1.2.2 (Zhang et al. 2020). Phylogenetic trees were constructed with both maximum likelihood (ML) and Bayesian inference (BI) methods. The ML method was conducted in IQ-TREE v. 2.0.4 (Nguyen et al. 2015) with 10000 ultrafast bootstrap (UFB) replicates (Hoang et al. 2018) and was performed until a correlation coefficient of at least 0.99 was reached. The BI method was performed in MrBayes v. 3.2.1 (Ronquist et al. 2012), and the best-fit model was obtained based on the Bayesian information criterion computed with PartitionFinder v. 2.1.1 (Lanfear et al. 2017). In this analysis, the first, second, and third codons of Cyt*b*, COI, RAG1, IRBP, and EGR2B were defined.

The analysis suggested the best partition scheme for each codon position of Cyt*b*, COI, RAG1, IRBP, and EGR2B. As a result, SYM+G was selected as the best model for the first codon of Cyt*b*; HKY+I was selected as the best model for the second codon of Cyt*b*, COI, EGR2B, IRBP, and RAG1, and the first codon of EGR2B; TRN+I+G was selected as the best model for the third codon of Cyt*b*; TRNEF+G and TIM+G were selected as the best models for the first and third codons of COI; TVM was selected as the best model for the third codon of EGR2B; TVM+I was selected as the best model for the first codon of IRBP and RAG1; and K80+G was selected as the best model for the third codon of IRBP and RAG1. Two independent runs were conducted in BI analysis, each of which was performed for 2 × 10^7^ generations and sampled every 1,000 generations. The first 25% of the samples were discarded as burn-in, resulting in a potential scale reduction factor of < 0.01. Nodes in the trees were considered well supported when the Bayesian posterior probabilities (BPP) were ≥ 0.95 and the MLUFB value was ≥95%. Uncorrected *p*-distances (1000 replicates) based on Cyt*b* and COI were estimated using MEGA 7.0.

### ﻿Morphological data and comparisons

Morphometric data were collected from 11 well-preserved specimens of the new species (Table 3). A total of 37 measurements were recorded to the nearest 0.1 mm with digital calipers following the protocol of Kottelat (1984) and Liu et al. (2012a). All measurements are taken on the left side of the fish specimens. Measurements for the new species in this study are provided in Table 3. All pre-processing of morphological data was performed in Microsoft Excel (Microsoft Corporation 2016) and statistical analyses were performed using SPSS 21.0 (SPSS, Inc., Chicago, IL, USA). Comparative data for the 18 species of the genus *Balitora* were obtained from the literature (Table 4). Vertebrae were counted from x-ray scanned images and skeletal counts refer to the previous Kumkar et al. (2016a) and Min et al. (2022).

**Table 3.** Morphological characters and measurement data (mm) of the new species *Balitoraanlongensis* sp. nov. described in this study.

Characters	GZNU2023 0215007	GZNU2023 0215008	GZNU2023 0215009	GZNU2023 0215010	GZNU2023 0215011	GZNU2023 0215012	GZNU2023 02150113	GZNU2023 0215014	GZNU2023 0215015	GZNU2023 0215016	GZNU2023 0215017	Range	Mean ± SD
Dorsal-fin rays	iii, 8½	iii, 8½	iii, 8½	iii, 8½	iii, 8½	iii, 8½	iii, 8½	iii, 8½	iii, 8½	iii, 8½	iii, 8½	/	/
Pectoral-fin rays	viii, 11	viii, 11	viii, 11	viii, 11	viii, 11	viii, 11	viii, 11	viii, 11	viii, 11	viii, 11	viii, 11	/	/
Pelvic-fin rays	ii, 9	ii, 9	ii, 9	ii, 9	ii, 9	ii, 9	ii, 9	ii, 9	ii, 9	ii, 9	ii, 9	/	/
Anal-fin rays	iii, 5½	iii, 5½	iii, 5½	iii, 5½	iii, 5½	iii, 5½	iii, 5½	iii, 5½	iii, 5½	iii, 5½	iii, 5½	/	/
Caudal-fin rays	17	17	17	17	17	17	17	17	17	17	17	/	/
Lateral-line pores/scales	68	68	68	68	67	68	68	67	66	68	68	66–68	/
Total length	60.4	57.7	51.7	56.9	52.2	59.6	56.7	53.6	49.7	60.4	55.8	49.7–60.4	55.9 ± 3.7
Standard length	47.9	44.6	42.4	44.7	42.3	47.7	45.9	42.5	39.8	48.2	44.3	39.8–48.2	44.6 ± 2.7
Body depth	5.2	5.3	5.6	5.5	4.7	5.8	5.6	5.0	4.5	5.8	5.1	4.5–5.8	5.3 ± 0.4
Body width	6.5	5.4	5.8	5.2	5.3	6.0	5.9	5.4	5.1	6.4	5.3	5.1–6.5	5.7 ± 0.5
Head length	10.3	10.0	9.3	10.6	9.4	10.5	10.4	9.9	8.4	9.9	9.9	8.4–10.6	9.9 ± 0.6
Head depth	4.9	4.2	4.3	4.3	4.2	4.1	4.0	3.8	3.6	4.5	4.1	3.6–4.9	4.2 ± 0.3
Head width	8.7	6.8	7.0	6.7	6.6	7.2	7.2	6.1	6.3	7.0	6.9	6.1–8.7	7.0 ± 0.7
Pre-anterior distance	3.5	4.3	3.4	3.8	3.7	4.6	3.6	3.9	3.8	3.9	4.4	3.4–4.6	3.9 ± 0.4
Distance between anterior nostrils	2.8	2.9	2.8	3.1	2.6	3.0	2.9	2.3	2.1	2.4	2.1	2.1–3.1	2.6 ± 0.4
Distance between posterior nostrils	3.8	3.7	3.6	3.6	3.8	3.4	3.6	3.2	2.9	3.4	3.1	2.9–3.8	3.5 ± 0.3
Distance between anterior and posterior nostrils	2.0	1.7	1.1	1.6	1.5	1.6	1.3	1.3	1.4	1.5	1.3	1.1–2.0	1.5 ± 0.2
Snout length	5.9	5.3	5.2	5.6	5.2	6.1	5.4	5.1	4.9	5.6	5.6	4.9–6.1	5.4 ± 0.4
Upper jaw length	2.8	2.5	2.6	2.5	2.5	2.8	2.1	2.7	2.3	2.8	2.6	2.1–2.8	2.6 ± 0.2
Lower jaw length	2.0	1.8	1.6	1.8	2.0	1.9	1.4	1.9	1.4	2.1	2.0	1.4–2.1	1.8 ± 0.2
Mouth width	3.6	3.8	3.5	3.8	4.1	4.2	4.0	3.6	3.4	4.0	3.7	3.4–4.2	3.8 ± 0.3
Eye diameter	1.3	1.1	1.0	0.9	1.0	0.9	0.8	0.9	0.8	1.1	0.9	0.8–1.3	1.0 ± 0.1
Interorbital distance	4.7	4.4	4.4	4.4	4.1	4.7	3.5	3.7	3.9	4.3	3.7	3.5–4.7	4.2 ± 0.4
Predorsal length	22.3	21.2	19.2	20.9	19.5	21.9	21.0	20.1	18.2	22.0	20.5	18.2–22.3	20.6 ± 1.3
Dorsal-fin base length	7.4	6.0	6.2	6.3	5.3	7.0	6.7	6.7	6.1	7.7	6.4	5.3–7.7	6.5 ± 0.7
Dorsal-fin length	10.9	10.5	9.6	10.3	10.2	10.4	10.1	10.0	8.9	11.0	10.1	8.9–11.0	10.2 ± 0.6
Pectoral-fin length	11.7	10.4	10.0	10.9	9.8	11.1	11.6	9.9	10.2	11.8	11.2	9.8–11.8	10.8 ± 0.8
Pectoral-fin base length	4.9	4.0	3.8	3.4	3.8	4.2	4.1	3.3	4.0	4.7	4.3	3.3–4.9	4.0 ± 0.5
Pre-pectoral length	9.3	8.7	7.3	8.2	7.2	8.4	7.8	8.2	7.4	8.1	7.8	7.2–9.3	8.0 ± 0.6
Pelvic-fin length	10.0	10.1	8.5	9.4	8.8	10.4	9.8	9.2	8.5	10.3	9.2	8.5–10.4	9.5 ± 0.7
Pelvic-fin base length	3.4	2.6	3.2	2.6	2.6	2.8	2.9	2.9	2.7	2.9	2.6	2.6–3.4	2.8 ± 0.3
Pre-pelvic length	22.6	21.7	19.7	20.1	20.2	22.7	21.5	20.3	18.8	21.8	20.1	18.8–22.7	20.9 ± 1.3
Anal-fin length	8.7	8.5	7.3	8.4	7.6	8.5	8.0	7.9	6.9	8.6	7.8	6.9–8.7	8.0 ± 0.6
Anal-fin base length	3.9	3.4	3.6	3.8	3.4	3.3	4.0	3.8	3.3	3.6	3.4	3.3–4.0	3.6 ± 0.3
Pre-anal length	34.8	33.0	30.4	32.5	31.6	35.3	33.5	31.2	29.8	35.2	31.9	29.8–35.3	32.7 ± 1.9
Distance between origin of pectoral fin and origin of ventral fin	10.0	9.4	9.0	9.8	9.3	10.8	10.2	9.1	8.2	10.7	9.7	8.2–10.8	9.7 ± 0.8
Distance between origin of ventral fin and origin of Anal fin	10.6	8.9	8.8	9.2	10.0	10.0	10.0	8.3	8.4	10.3	9.5	8.3–10.6	9.5 ± 0.8
Distance between end of Anal fin and anus	1.8	2.4	2.4	2.2	2.9	3.0	2.3	1.9	1.7	2.4	2.1	1.7–3.0	2.3 ± 0.4
Caudal peduncle length	8.6	9.6	8.5	8.7	7.5	9.6	9.1	8.1	7.8	9.9	9.2	7.5–9.9	8.8 ± 0.8
Caudal peduncle depth	3.1	2.9	3.1	2.7	2.7	3.1	2.8	3.0	2.3	3.2	3.0	2.3–3.2	2.9 ± 0.3
Inner maxillary barbel length	1.4	2.0	1.4	2.1	1.7	2.5	1.5	2.4	1.7	2.0	1.5	1.4–2.5	1.8 ± 0.4
Outer maxillary barbel length	1.1	1.0	1.2	0.9	0.9	1.5	1.0	1.2	0.9	1.1	0.9	0.9–1.5	1.1 ± 0.2
Inner rostral barbel length	1.4	1.1	1.0	1.2	1.1	1.6	1.3	1.4	1.1	1.6	1.1	1.0–1.6	1.3 ± 0.2
Outer rostral barbel length	1.9	2.0	1.7	2.0	1.9	1.8	1.9	1.8	1.8	2.2	1.7	1.7–2.2	1.9 ± 0.1

**Table 4.** Comparison of the diagnostic characters of the new species described here with those selected for the 16 species of the genus *Balitora*. Gray shading indicates clear differences in characters compared to *Balitoraanlongensis* sp. nov.

ID	Species	Maxillary barbels	Dorsal fin rays	Anal fin rays	Pectoral fin rays	Pelvic fin rays	Lateral-line scales	Dorsal black spot	Pectoral fin tip	Position of origin of dorsal-fin and origin of pelvic-fin	Tip of pelvic fin reaching anus
1	*Balitoraanlongensis* sp. nov.	2	iii, 8½	iii, 5½	viii, 11	iii, 9	66–68	6–7	Not reaching to pelvic fin origin	Anterior to the pelvic-fin origin	Yes
2	*B.annamitica*	1	–	–	viii–x, ?	–	61–62	–	–	–	–
3	*B.brucei*	1	iii, 8	i, 5	ix, 11	ii, 9	65–69	–	–	–	–
4	*B.burmanica*	1	iii, 8	i, 5	ix, 11	ii, 8	70–72	–	–	–	–
5	*B.chipkali*	1	iii, 8	iii, 5	iii–ix, 11–12	ii, 9	66–68	7	Not reaching to pelvic fin origin	Opposite to pelvic-fin origin	Yes
6	*B.eddsi*	1	iii, 9	iii, 5\7	vi, 10–12	ii, 8–9	66–67	Without	Not reaching to pelvic fin origin	Slightly anterior to pelvic-fin origin	NO
7	*B.jalpalli*	1	iii, 8	ii, 5	ix, 10–11	ii, 8–9	64–66	9	Not reaching to pelvic fin origin	Opposite to pelvic-fin origin	NO
8	*B.kwangsiensis*	2	iii, 8	ii, 5	vi–viii, 10–13	ii, 8	61–65	6–8	Not reaching to pelvic fin origin	Anterior to the pelvic-fin origin	Yes
9	*B.lancangjiangensis*	1	iii, 8	ii, 5	viii, 10–12	ii, 8–9	68–70	7–8	Not reaching to pelvic fin origin	Opposite to pelvic-fin origin	NO
10	*B.laticauda*	1	iii, 8	iii, 5	viii–ix, 10–11	ii, 8–9	66–68	10	Not reaching to pelvic fin origin	Opposite to pelvic fin origin	NO
11	*B.longibarbata*	2	iii, 8	ii, 5	viii–x, 11–14	ii, 9–11	74–76	8–9	Not reaching to pelvic fin origin	Opposite to pelvic-fin origin	NO
12	*B.ludongensis*	2	iii, 8	ii, 5	vi–vii, 11–12	ii, 6–7	69–74	6–9	Not reaching to pelvic fin origin	Slightly anterior to pelvic-fin origin	Yes
13	*B.meridionalis*	1	–	–	–	–	–	–	–	–	–
14	*B.mysorensis*	1	iii, 8–9	ii, 5	viii-ix 10–12	ii, 8–9	68–69	–	–	–	–
15	*B.nantingensis*	1	iii, 8	ii, 5	viii–x, 9–12	ii, 9	59–64	–	Not reaching to pelvic fin origin	Slightly anterior to pelvic-fin origin	–
16	*B.elongata*	1	iii, 8	ii, 5	x, 10–12	iii, 8	67	7	Not reaching to pelvic fin origin	Opposite to pelvic-fin origin	NO
17	*B.tchangi*	1	iii, 7	ii, 5	xii, 13	v, 13	74	8	Beyond the origin of pelvic fin	Posterior to the pelvic-fin origin	NO

## ﻿Results

### ﻿Phylogenetic analyses and genetic divergence

**Figure 3.**
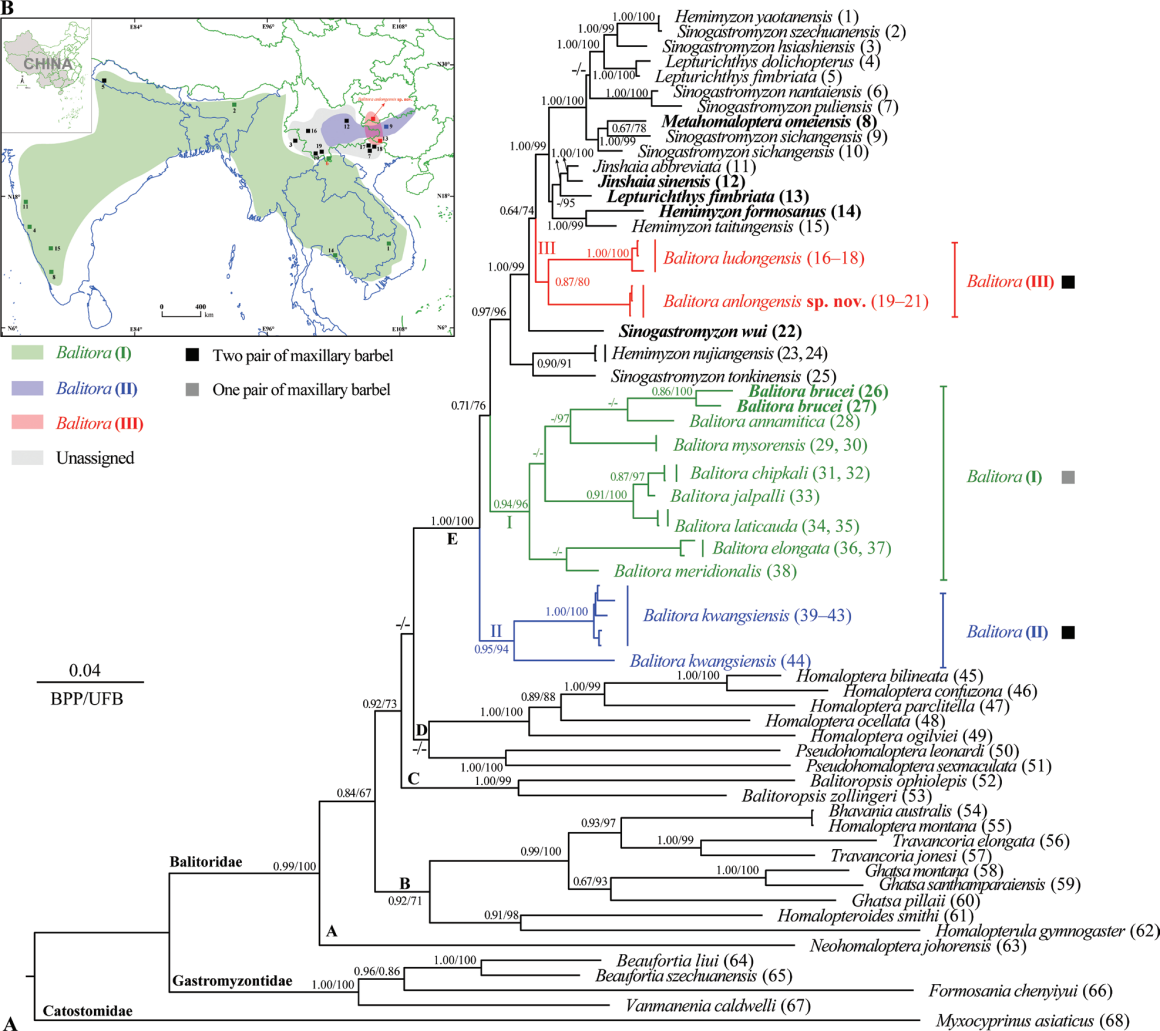
**A** Distributions of the different clades of the genus *Balitora* in Asia **B** phylogenetic tree reconstructed based on combined two mitochondrial (Cyt*b* and COI) and three nuclear gene (RAG1, IRBP, and EGR2B) fragments. In this phylogenetic tree, ultrafast bootstrap (UFB) supports from maximum likelihood (ML) analyses/Bayesian posterior probabilities (BPP) from Bayesian inference (BI) analyses are noted beside nodes. The scale bar represents 0.04 nucleotide substitutions per site. The numbers at the tips of branches correspond to the ID numbers in Table 2.

Both ML and BI phylogenies were constructed based on two mitochondrial and three nuclear genes, with a sequence length of 5382 base pairs. The BI and ML phylogenetic trees show a highly consistent topology that strongly supports the monophyly of the family Balitoridae and can be divided into five major clades (Fig. 3).

The phylogenetic tree shows that *Balitora* is not a monophyletic and contains three distant clades: Clade I, including *B.meridionalis* Kottelat, 1988, *B.elongata*, *B.laticauda* Bhoite, Jadhav & Dahanukar, 2012, *B.jalpalli* Raghavan, Tharian, Ali, Jadhav & Dahanukar, 2013, *B.chipkali* Kumar, Katwate, Raghavan & Dahanukar, 2016, *B.mysorensis* Hora, 1941, *B.annamitica* Kottelat, 1988, and *B.brucei* Gray, 1830 (type species of the genus *Balitora*); Clade II, containing only *B.kwangsiensis*; and Clade III, including *B.ludongensis* and the new species described in this study (Fig. 3). In addition, this study also found the non-monophyly of *Hemimyzon*, *Sinogastromyzon*, and *Lepturichthys* Regan, 1911 (Fig. 3). The smallest uncorrected *p*-distances between *Balitoraanlongensis* sp. nov. and other species of the genus *Balitora* were 7.3% in Cyt*b* (with *B.ludongensis*) and 9.2% in COI (with *B.mysorensis*) (Tables 5, 6). This is greater than the mitochondrial genetic differences with other known species, for example, 4.5% in Cyt*b* between *B.chipkali* and *B.laticauda* and 2.1% in COI between *B.jalpalli* and *B.laticauda* (Tables 5, 6).

**Table 5.** Uncorrected *p*-distance (%) between eight species of the genus *Balitora* based on mitochondrial Cyt*b*.

ID	Species	1	2	3	4	5	6	7
1	*Balitoraanlongensis* sp. nov.							
2	*Balitoraludongensis*	7.3						
3	*Balitorabrucei*	10.4	9.5					
4	*Balitorachipkali*	12.0	11.9	9.9				
5	*Balitoraelongata*	9.2	9.5	10.7	11.1			
6	*Balitorakwangsiensis*	9.5	9.0	9.6	12.8	10.5		
7	*Balitoralaticauda*	10.9	10.6	8.3	4.5	10.3	11.4	
8	*Balitoramysorensis*	10.6	10.3	9.0	11.7	10.3	11.3	10.4

**Table 6.** Uncorrected *p*-distance (%) between nine species of the genus *Balitora* based on mitochondrial COI.

ID	Species	1	2	3	4	5	6	7	8
1	*Balitoraanlongensis* sp. nov.								
2	*Balitoraludongensis*	10.5							
3	*Balitorabrucei*	10.2	11.1						
4	*Balitorachipkali*	10.5	11.1	9.4					
5	*Balitorajalpalli*	10.3	11.3	9.7	1.1				
6	*Balitorakwangsiensis*	12.1	11.7	12.2	12.3	12.8			
7	*Balitoralaticauda*	11.1	11.7	9.3	2.5	2.1	12.5		
8	*Balitoramysorensis*	9.2	10.0	6.8	9.0	8.6	11.5	8.4	
9	*Balitoranujiangensis*	9.6	10.0	9.4	9.6	9.8	10.0	9.8	8.4

Thus, the population at this locality represents an independently evolved lineage and is described as a new species, *Balitoraanlongensis* sp. nov., below.

### ﻿Taxonomic account

#### ﻿ Balitora anlongensis

Taxon classificationAnimaliaCypriniformesBalitoridae

Luo, Chen, Zhao, Yu, Lan & Zhou sp. nov.

02707D53-F8B5-531F-9B2E-119FC564E2E9

https://zoobank.org/C1E6B69D-57B1-4AC3-ACCC-3A92F6E2E653

**Figure 4.**
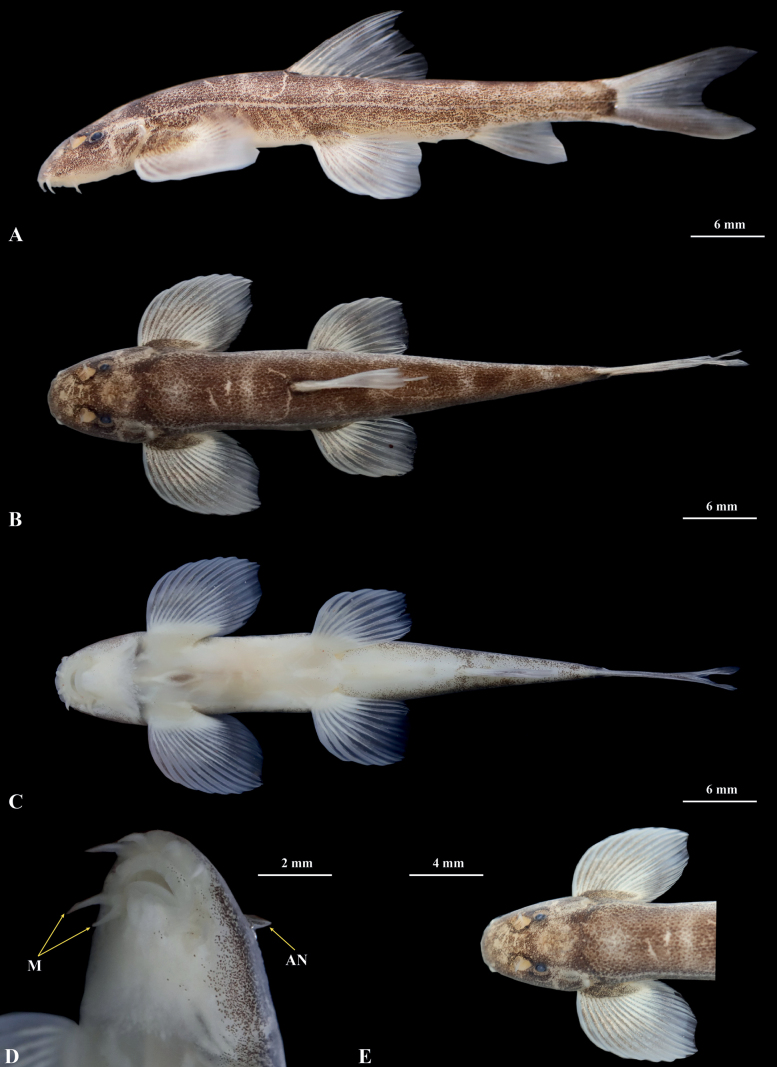
Morphological characters of holotype GZNU20230215007 of *Balitoraanlongensis* sp. nov. in preservative (10% formalin) **A** lateral view **B** dorsal view **C** ventral view **D** ventral side view of head, and **E** dorsal side view of head. Photos from Tao Luo. Abbreviations: M, maxillary barbels; AN, anterior nostril.

**Figure 5.**
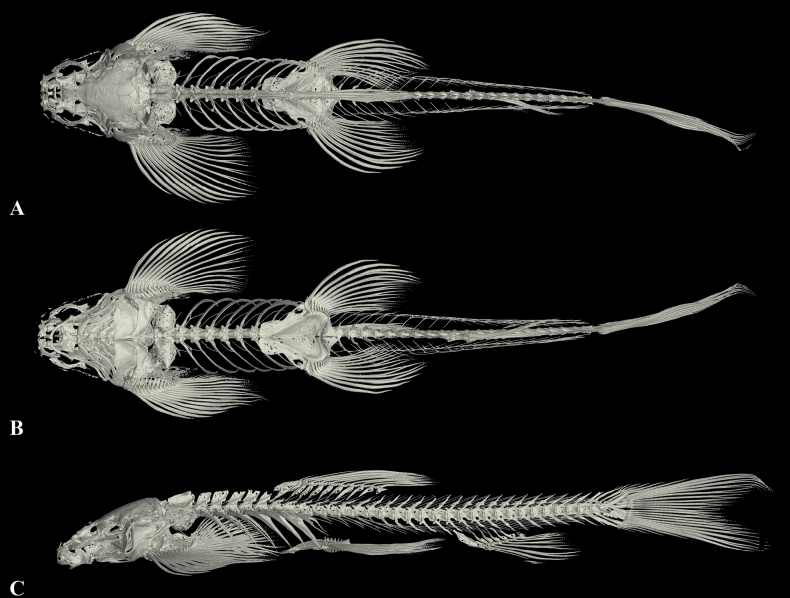
Three-dimensionally reconstructed model of the skeleton of *Balitoraanlongensis* sp. nov. (paratype GZNU20230223017, standard length 44.3 mm) **A** dorsal view **B** ventral view, and **C** lateral view.

**Figure 6.**
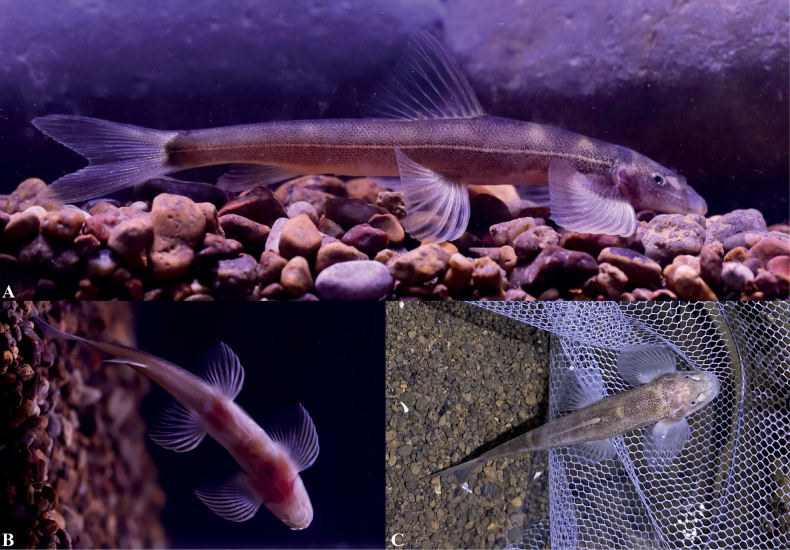
*Balitoraanlongensis* sp. nov. in life, paratypes GZNU20230106001 (photos **A** and **B**) and GZNU20230215014 (photo C) **A** right-side view **B** ventral side view, and **C** dorsal view. Photographs **A, B** were shot indoors at ~ 9:00 p.m. Photo **C** was taken in the cave at ~ 15:00 noon.

Figs 4 , 5 , 6 , Table 3

##### Type material.

***Holotype*.** GZNU20230215007 (Fig. 4), 60.4 mm total length (TL), 47.9 mm standard length (SL), collected by Tao Luo on 15 February 2023 in Xīniú Cave (犀牛洞), NaNao Village, Xinglong Town, Anlong County, Guizhou Province, China (105.59143424E, 25.07096446N, 1450 m. a.s.l.; Figs 1, 2).

***Paratypes*.** Ten specimens from the same locality as the holotype: GZNU20230215008–215017, collected by Tao Luo, Xin-Rui Zhao, Wei-Feng Wang, Jing Yu, and Chang-Ting Lan on 15 February 2023.

##### Comments.

The new species is assigned to the genus *Balitora* based on the combination of the following diagnostic characters (Kottelat 1988; Kottelat and Chu 1988): (1) body strongly depressed; head and abdomen ventrally flattened; (2) mouth inferior, arched, with both jaws covered by a horny sheath; (3) rostral flap divided into three lobes, both lips with one or two rows of papillae, lower lip not interrupted; (4) one or two pairs of maxillary barbels; (5) gill-openings extending on the ventral surface of head; (6) unbranched pelvic rays two, 8–10 unbranched pectoral rays and 10–12 branched pectoral rays; and (7) adhesive pads present on ventral surface of the 8–11 anterior most pectoral rays and 3–4 anterior most pelvic rays.

##### Diagnosis.

*Balitoraanlongensis* sp. nov. can be distinguished from other congeners by the following combination of characters: (1) two pairs of maxillary barbels; (2) dorsal fin rays iii, 8½; (3) pectoral fin viii, 11; (4) pelvic fin rays ii, 9; (5) anal fin rays iii, 5½; (6) lateral-line scales 66–68; (7) tip of pectoral fin not reaching to the pelvic fin origin; (8) dorsal fin origin anterior to the pelvic fin origin; (9) tip of the pelvic fin reaching to the anus; (10) eyes small, eye diameter equal to outer maxillary barbel length; (11) six to seven indistinctly separated transversely oval blotches on the dorsal side; and (12) each fin transparent and unpigmented in life.

##### Description.

Morphological data of the 11 specimens of the *Balitoraanlongensis* sp. nov. are provided in Table 3. Body elongated and sub-cylindrical, posterior portion gradually compressed from dorsal fin to caudal-fin base, with deepest body depth anterior to dorsal-fin origin, deepest body depth 11–13% of SL. Dorsal profile slightly convex from snout to dorsal-fin insertion, then straight from posterior portion of dorsal-fin origin to caudal-fin base. Pelvic profile flat. Head blunt and depressed, head length (HL) 21–24% of SL and greater than head width, head width greater than depth (head width/head depth = 1.7). Snout short, oblique, and blunt, length 52–58% HL. Interorbital space wide and flat. Mouth inferior, small and curved, mouth corner situated below anterior nostril, upper and lower lips smooth and fleshy. Relatively shallow preoral groove present between rostral cap and upper lip, extending across corners of mouth. Mouth width 35–44% of head width. Rostral cap around upper lip divided into three lobes, median one largest, slightly curved. Four pairs of barbels: two pairs of rostral barbels, short, outer rostral barbel longer than inner one; two pairs of maxillary barbels, short, situated at corner of mouth: outer maxillary barbels longer than inner one. Upper and lower lips connected at corner of mouth, upper lip with 3–5 papillae in middle, and lower lip thin, with large fleshy papilla at upper and lower lip joint. Lower jaw with radiate ridges on its surface. Two longitudinal fleshy ridges on mid-chin. Anterior and posterior nostrils closely set, anterior nostril without any elongated barbel-like tip. Eyes reduced, 8–13% HL. Gill opening large, extending to origin of pectoral fin, gill rakers absent.

Dorsal fin rays iii, 8½, pectoral fin rays viii,11, pelvic fin rays ii, 9, anal fin rays iii, 5½, and 17 branched caudal fin rays. Dorsal fin long, 22–24% of SL, nearly equal to head length, distal margin truncated, origin anterior to pelvic fin insertion, situated slightly anterior to midpoint between snout tip and the caudal fin base, first branched ray longest, shorter than HL, tip of the dorsal fin extending to the vertical of the anus. Pectoral fin elongated and developed, distal margin rounded, pectoral fin length slightly greater than HL, 23–25% of SL, tip of the pectoral fin extends backward beyond 3/4 of the distance between the origin of the pectoral fin and the origin of the pelvic fin, without reaching to the pelvic fin-origin. Pelvic fin moderately developed, distal margin rounded, pectoral fin length approximately equal to HL, 23–26% of SL, vertically aligned with the third branched ray of the dorsal fin, pelvic fin origin closer to the snout tip than the caudal fin base and closer to the anal fin origin than the snout tip, tips of the pelvic fin reaching to the anus. Anus ~ 4/5 distance from posterior end of the pelvic fin base to the anal fin origin. Anal fin short, 17–19% of SL, distal margin truncated, origin close to the anus and far from the caudal fin base, spacing ~ 2.9 mm, tips of the anal fin extending backwards and not reaching caudal fin base, distance between the end of the anal fin and the anus 5.4-times the eye diameter. Caudal fin deeply forked, upper lobe equal in length to the lower one, tips pointed, caudal peduncle length 8.8 mm, caudal peduncle depth 2.9 mm, without adipose crests along both dorsal and ventral sides. Vertebrae 36: nine pre-dorsal abdominal, 19 abdominal (including four Weberian and 15 pre-dorsal ones), and 17 caudal (including three pre-anal caudal and the hypural complex) (Fig. 5).

Body smooth, covered with thin scales all over except for on the ventral side, head, and fins. Lateral line complete and straight, with 66–68 lateral line scales, exceeding the tip of the pectoral fin and reaching the base of the caudal fin. Two air bladder chambers, posterior chamber of the air bladder slightly developed and closed (Fig. 5).

##### Coloration.

Dwelling in the water bodies of the cave (Fig. 6), dorsal and lateral sides of the body grey-brown, ventral side white, slightly pinkish. Dorsal side of the head grayish-white, grayish-brown pigmented spots predominate. Dorsal side of the body with six to seven indistinctly transversely oval blotches separated by grayish-yellow gaps, and usually two blotches anterior to the dorsal fin origin, one to two blotches at the dorsal fin base, and three to four blotches posterior to the dorsal fin end. Except for discontinuous black spots on the distal end of the caudal fin, the remaining fins are transparent and unpigmented. Base of the caudal fin deeply pigmented. Lateral line of the body from the posterior to the eye to the base of the caudal fin light grayish yellow. After being moved from inside the cave to outside, the body pigmentation deepened in ~ 4 hrs. After being fixed in 10% formalin and stored (Fig. 4), the body pigmentation deepened and there was pigmentation on the unbranched fins of each fin.

##### Sexual dimorphism.

No sexual dimorphism was observed based on the present specimens of *Balitoraanlongensis* sp. nov.

##### Comparisons.

Comparative data of *Balitoraanlongensis* sp. nov. with 19 species within the genus *Balitora* are given in Table 4.

*Balitoraanlongensis* sp. nov. differs from *B.annamitica*, *B.brucei*, *B.burmanica* Hora, 1932, *B.chipkali*, *B.eddsi* Conway & Mayden, 2010, *B.jalpalli*, *B.lancangjiangensis*, *B.laticauda*, *B.meridionalis*, *B.mysorensis*, *B.nantingensis* Chen, Cui & Yang, 2005, *B.elongata*, and *B.tchangi* based on the presence of two maxillary barbels at each corner of the mouth (vs one maxillary barbel at each corner of the mouth). *Balitoraanlongensis* sp. nov. can be further distinguished from *B.eddsi* and *B.tchangi* based on dorsal fin rays (iii, 8½ vs iii, 9 in *B.eddsi* and iii, 7 in *B.tchangi*); from *B.jalpalli*, *B.lancangjiangensis*, *B.laticauda*, *B.nantingensis*, *B.elongata*, and *B.tchangi* by the tip of pelvic fin reaching to the anus (vs not reaching to the anus); from *B.brucei* by pectoral fin rays (viii, 11 vs ix, 11); and from *B.jalpalli*, *B.lancangjiangensis*, *B.laticauda*, *B.elongata*, and *B.tchangi* by the dorsal fin origin being anterior to the pelvic fin origin (vs opposite/posterior to pelvic fin origin; see Table 4 for detailed comparisons).

*Balitoraanlongensis* sp. nov. and *B.kwangsiensis*, *B.longibarbata*, and *B.ludongensis* are distributed in the Pearl River and share two maxillary barbels at each corner of the mouth, but can be distinguished by the combination of serial characters. *Balitoraanlongensis* sp. nov. can be distinguished from *B.kwangsiensis* by anal fin rays (iii, 5½ vs ii, 5), pelvic fin rays (ii, 9 vs ii, 8), lateral-line scales (66–68 vs 61–65), and each fin being transparent and unpigmented in life (vs each fin having black spots); from *B.longibarbata* by the pelvic fin rays (ii, 8 vs ii, 9–11), anal fin rays (iii, 5½ vs ii, 5), lateral-line scales (66–68 vs 74–76), the dorsal fin origin being anterior to the pelvic fin origin (vs opposite to the pelvic fin origin), and the tip of the pelvic fin reaching to the anus (vs not reaching to the anus).

Phylogeny constructed based on combined mitochondrial and nuclear genes shows that *Balitoraanlongensis* sp. nov. is close to *B.ludongensis* and can be distinguished by the combination of the following morphological characters: eight unbranched pectoral fin rays (vs six to seven), anal fin rays (iii, 5½ vs ii, 5), pelvic fin rays (ii, 9 vs ii, 6–7), lateral-line scales (66–68 vs 69–74), dorsal fin origin far anterior to the pelvic fin origin (vs slightly anterior to the pelvic fin origin), body depth 13–14% of the SL (vs 15–20%), body width 12–14% of SL (vs 18–21%), head depth 38–48% of HL (vs 51–67%), head width 109–134% of body width (vs 87–107%), transversely oval blotches on dorsal side indistinctly separated (vs distinctly separated), and each fin transparent and unpigmented in life (vs each fin having black spots).

##### Distribution and ecology.

*Balitoraanlongensis* sp. nov. is only known from the type locality, a vertical cave some distance from NaNao Village, Xinglong Town, Anlong County, Guizhou Province, China at an elevation of 1387 m (Figs 1, 2). The type locality is located in the Nanpanjiang River, a tributary of the Pearl River. There is no surface stream outside the cave. The cave is ~ 50 m long and has a small volume of water during dry periods, but is a source of domestic water for the local population. Within this cave, *Balitoraanlongensis* sp. nov. co-occurred with fish (*Triplophysa* sp. and *Misgurnusanguillicaudatus*), frog (*Odorrana* sp.), red-eared slider (*Trachemysscripta*), and crab (*Diyutamoncereum*) species. Outside the cave, the arable land was farmed to produce maize, wheat, and potatoes.

##### Etymology.

The specific epithet “*anlongensis*” is in reference to the type locality of the new species: NaNao Village, Xinglong Town, Anlong County, Guizhou Province, China. We propose the common English name “Anlong stone loach” and the Chinese name “ān lóng Pá Qīu (安龙爬鳅)”.

## ﻿Discussion

This work described a new cavefish species from Xinglong Town, Anlong County, Guizhou, named *Balitoraanlongensis* sp. nov., based on morphological comparisons (see the comparison above) and genetic differences (Fig. 3; Tables 3, 4), i.e., forming a separate lineage and with an uncorrected *p*-distance of 7.3% (in Cyt*b*) from *B.ludongensis*. This evidence supports the validity of the new species and indicates that this species is the first cave fish within the genus *Balitora* in China. The description of this new species increases the number of species of the genus *Balitora* from 19 to 20 (Table 1), with the number known from Guizhou increasing to two species (Zheng 1989), i.e., *B.kwangsiensis* and *Balitoraanlongensis* sp. nov. Notably, known species of the genus *Balitora* are mainly distributed in southern India, Nepal, Thailand, Vietnam, and in Yunnan, Northwestern Guangxi, and Southeastern Guizhou in China (Fricke et al. 2023) (Fig. 1). The discovery of this new species for the first time in southwestern Guizhou and the discontinuous distribution of *Balitora* in the Nanpanjiang and Hongshui River basins (Fig. 2) suggest that there may be undescribed species within these regions. In other words, the species diversity of *Balitora* in Southwestern China may be underestimated and needs to be fully and systematically surveyed.

The genus *Balitora* may need to be revised in the future by combining evidence from phylogenetic and morphological evidence. The taxonomy of the genus *Balitora* based on morphological characters has undergone two major revisions, with the main controversy being over its key diagnostic characteristics, i.e., one pair of maxillary barbel (Chen 1978; Chen and Tang 2000) or two unbranched pelvic fin rays (Kottelat and Chu 1988). In the phylogenetic tree reconstructed in this study based on two mitochondrial and three nuclear genes, *Balitora* was divided into four distant clades, supporting the non-monophyletic results of *Balitora* considered in previous studies (Kumkar et al. 2016a). In addition, within the genus *Balitora*, the number of maxillary barbels (one or two) does not have strict phylogenetic correspondence and therefore may need to be revised and supplemented for key diagnoses (Fig. 3A). Clade I contains the type species *B.brucei*, and therefore Clade I is the true *Balitora*, i.e., *Balitorasensu stricto*, which is mainly distributed in India, Nepal, Thailand, and Vietnam (green area in Fig. 3B). Clade II contains the type species of *Sinohomaloptera*, *S.kwangsiensis*, thus supporting the validity of *Sinohomaloptera*, and is mainly distributed in northern Guangxi, southeastern Guizhou, and eastern Yunnan, China (blue area in Fig. 3A). The remaining clade III may represent a new taxonomic group, with Clade III mainly distributed in the Zuojiang River basin in western Guangxi and the Nanpanjiang River basin in southwestern Guizhou (red area in Fig. 3B). The discovery of this new species in the Nanpanjiang River basin of Guizhou extended the current distribution of *Balitora* to the northeast. However, this work failed to complete the revision of Clade III of *Balitora* because morphological evidence of sufficient specimens was not obtained. Thus, to complete this revision, in the future, sufficient specimens, skeletal CT, and chromosomal, anatomical, and genomic-level phylogenetic relationships can be obtained to address this revision.

### ﻿Comparative material examined

*Balitoraludongensis* (*n* = 2): China: Guangxi: Jingxi County: Ludong Town (type locality): GZNU 20230207001–0207002.

*Balitorakwangsiensis* (*n* = 2): China: Yunnan: Amojiang River basin: GZNU20230226001–0226002.

## Supplementary Material

XML Treatment for Balitora anlongensis

## ﻿Additional information

### Author ORCIDs

Tao Luo https://orcid.org/0000-0003-4186-1192

Zhi-Xia Chen https://orcid.org/0009-0002-2066-494X

Xin-Rui Zhao https://orcid.org/0000-0002-9125-6276

Jing Yu https://orcid.org/0009-0004-3629-3826

Chang-Ting Lan https://orcid.org/0009-0007-2381-3601

Jia-Jun Zhou https://orcid.org/0000-0003-1038-1540

Ning Xiao https://orcid.org/0000-0002-7240-6726

Jiang Zhou https://orcid.org/0000-0003-1560-8759

### Data availability

All of the data that support the findings of this study are available in the main text or Supplementary Information.

## Supplementary materials

Supplementary material 1 List of cavefishes in China Data type xlsx https://binary.pensoft.net/file/943525This dataset is made available under the Open Database License (http://opendatacommons.org/licenses/odbl/1.0/). The Open Database License (ODbL) is a license agreement intended to allow users to freely share, modify, and use this Dataset while maintaining this same freedom for others, provided that the original source and author(s) are credited.Tao Luo, Zhi-Xia Chen, Xin-Rui Zhao, Jing Yu, Chang-Ting Lan, Jia-Jun Zhou, Ning Xiao, Jiang Zhou

Supplementary material 2 Specific primers used to amplify two mitochondrial and three nuclear genes Data type xlsx https://binary.pensoft.net/file/943526This dataset is made available under the Open Database License (http://opendatacommons.org/licenses/odbl/1.0/). The Open Database License (ODbL) is a license agreement intended to allow users to freely share, modify, and use this Dataset while maintaining this same freedom for others, provided that the original source and author(s) are credited.Tao Luo, Zhi-Xia Chen, Xin-Rui Zhao, Jing Yu, Chang-Ting Lan, Jia-Jun Zhou, Ning Xiao, Jiang Zhou
